# The unnecessary workups and admissions of adolescents and young adults with spontaneous pneumomediastinum

**DOI:** 10.1038/s41598-024-55134-1

**Published:** 2024-02-24

**Authors:** Lindsay Wald, Celeste Yergin, Robin Petroze, Shawn Larson, Saleem Islam

**Affiliations:** Division of Pediatric Surgery, Department of Surgery, University of Florida College of Medicine, 1600 SW Archer Road, PO Box 100119, Gainesville, FL 32610 USA

**Keywords:** Diagnosis, Health care economics, Health services, Medical imaging, Paediatrics

## Abstract

Spontaneous pneumomediastinum (SPM) is a rare condition in children and young adults that raises concern for esophageal perforation or extension of an air leak, resulting in admissions with multiple interventions performed. To assess our outcomes, and to evaluate our resource utilization, we reviewed our experience with SPM. We conducted a retrospective review of SPM cases in patients aged 5–25 years old occurring between 2011 and 2021 at a single academic tertiary care center. Clinical, demographic, and outcome variables were collected and analyzed, and cohorts were compared using Fischer’s Exact Test and Welch’s T Test. 166 SPM cases were identified—all of which were Emergency Department (ED) presentations. 84% of the cases were admitted. 70% had Computerized Tomography (CT) scans, with no defined criteria for imaging. Comparison of floor admissions with discharges from the ED showed no significant difference in presenting symptoms, demographics, or outcomes between the two groups. Recurrence was noted in 4 patients with a range of 5.9 months–4.9 years from the initial episode. In the largest SPM study in the pediatric and young adult population, we noted no significant difference in management or outcomes in admitted or ED discharge patients nor those with CT imaging. Our results suggest that a large number of SPM can be managed safely with discharge from the ED.

## Introduction

Pneumomediastinum is a rare condition defined as the presence of extraluminal air in the mediastinum, and typically presents with retrosternal chest pain that radiates to the neck or back, occasionally with dyspnea, and dysphagia^1^. Feared, but rare sequalae of pneumomediastinum are cardiac tamponade and airway compression, which may require emergent exploration^2^. The condition is categorized into secondary, and spontaneous pneumomediastinum. Secondary pneumomediastinum is a result of traumatic or iatrogenic etiologies, such as endoscopic, post-operative, and airway-related procedures and may represent injury to the esophagus, tracheobronchial tree, or lungs. On the other hand, spontaneous pneumomediastinum (SPM) occurs with no clear inciting event despite some predispositions such as preexisting respiratory disease, excessive emesis, or lean body habitus^2,3^. In children, a severe asthma exacerbation is the major cause of SPM, and pediatric SPM has a bimodal incidence with those less than 7 (typically related to asthma) and then 13- to 17-year-old cases that are not due to asthma^4,5^.

Spontaneous cases have been known to have a benign course, with younger children’s issues related to the severity of an associated asthma exacerbation. These patients are usually subjected to a series of tests such as Computerized Tomography (CT) scans and even invasive procedures like endoscopic and bronchoscopic evaluations as well as inpatient admission for observation^5^. The argument for the current invasive management protocol is in concern for less benign causes of SPM, which may require additional diagnostic workup^8^. This escalation in diagnostic work-up is most often observed in patients with SPM presenting with multiple episodes of emesis in concern for Boerhaave syndrome, a life-threatening condition^6^.

In our current healthcare environment where costs have escalated and limited resources are being managed with increasing care, we considered the diminishing value of admission or even tests in patients with SPM. Creation of clinical pathways to help direct patient care can be helpful in standardizing interventions and have been successful in multiple other conditions. We hypothesized that a majority of cases of SPM can be discharged from the emergency department and clinical characteristics could predict the need for admission or additional investigations. To help understand and answer that question, this study was designed to collect and analyze data on all cases of SPM in children, adolescents, and young adults at our institution and to assess outcomes.

## Methods

### Patient identification and selection

Approval from the University of Florida Institutional Review Board (IRB) with exempt status (IRB202100306) was obtained prior to project initiation. A retrospective investigation of all pediatric and young adult cases of pneumomediastinum over a 10-year-period between 2011 and 2021 at the University of Florida was completed using ICD-9 and ICD-10 codes, in accordance with the guidelines and regulations set by the IRB. Patients with traumatic or iatrogenic causes of pneumomediastinum were excluded. SPM was defined as pneumomediastinum not secondary to chest wall trauma or medical procedures. The age range was limited to those between the ages of 5 and 25 at the time of their diagnosis to focus the investigation on the patient population that literature most commonly associates with SPM. University of Florida electronic medical records, via the institution’s Epic system, were used to acquire data. Due to the retrospective nature of the study, the need for informed consent was waived by University of Florida Institutional Review Board. Data were deidentified prior to analysis. The deidentified dataset analyzed in this study is available from the corresponding author on reasonable request.

### Clinical variables collected

Demographic, clinical, and outcome variables were recorded after comprehensive chart review. Demographic variables included age at time of onset, sex, race and ethnicity, and insurance status. Biracial patients were analyzed as “other.” Clinical variables included pre-existing conditions, activity at the time of onset, Body Mass Index (BMI), presentation location (Emergency Department (ED), outpatient), acute or gradual onset, symptoms, physical exam findings, vital signs (Blood Pressure (BP), Heart Rate (HR), Respiratory Rate (RR), temperature, and O_2_ saturation), laboratory evaluation with White Blood Cell (WBC) count and C-reactive Protein (CRP) level, imaging and imaging results, admission and discharge from ED, admitting service, level of admission, diagnostic testing, operative interventions and findings, medications prescribed, length of hospital stay, and return to ED within 30 days of initial visit. Outcomes variables included hospital complications (sepsis, respiratory distress, etc.), mortality, or morbidities. Additionally, charts were combed for mention of spontaneous pneumomediastinum in subsequent notes. Follow-up time was acquired and defined as the time from discharge to a provider appointment where spontaneous pneumomediastinum was discussed. Residual complaints at follow-up were gathered.

### Definitions

Recurrence was noted and defined as the number of days between the initial spontaneous pneumomediastinum presentation and the subsequent episode. In this definition, no threshold for minimum or maximum number of days was instituted. Gradual onset was defined as greater than 24 h from the time of symptom onset to initial presentation. Acute onset was defined as under 24 h from the time of symptom onset to initial presentation.

### Statistical analysis

After checking that the distribution of the data was not not significantly different from a normal distribution with the Shapiro–Wilk test, analyses were completed with descriptive statistical analysis, Welch’s T Test, One-way ANOVA, and Fisher Exact Test. All statistical analyses were performed using Minitab version 60 from Cary, NC. Figure was made using Graphpad Prism Version 9.3.1 for macOS, GraphPad Software, San Diego, California USA, www.graphpad.com.

### Ethics approval

This research study was conducted retrospectively from data obtained for clinical purposes. Use of data has been approved by the University of Florida Institutional Review Board (IRB202100306).

## Results

### Patient presentation

226 patients with pneumomediastinum were identified via ICD-9 and ICD-10 codes. After applying the exclusion criteria (those outside of 5–25 years old and iatrogenic or traumatic SPM etiology), 166 cases of spontaneous pneumomediastinum from 163 unique patients were included in this study. Table 1 provides the patient demographic information. All patients presented to the ED, with 63.9% experiencing an acute onset and 36.1% had a gradual onset of symptoms. The most common symptoms patients presented with were chest pain (71.7%), dyspnea (53%), and cough (38.6%). Table 2 summarizes the pertinent physical exam findings and symptoms experienced by patients at time of presentation at the ED. Of note, 25.3% of patients had no pertinent findings on physical exam, 18.7% were found to be wheezing, and 18.1% were found to have crepitus on chest wall or neck palpation. In addition, 64% of the patients reported a history of vomiting, while 38.6% had a medical history of asthma.

**Table 1:** Demographics of patients.

	n = 166
Median (IQR) age (years)	18 (15–21)
Sex, (%)	
Female	44 (27.2)
Male	118 (72.8)
Race, (%)	
Asian	1 (0.6)
Black	25 (15.1)
Hispanic	15 (9.0)
Native American	0 (0)
Native Hawaiian or Pacific Islander	0 (0)
White	112 (67.5)
Other	12 (7.2)
Median (IQR) height (cm)	171.5 (160.0–180.3)
Median (IQR) weight (kg)	63.5 (54.4–74.1)
Median (IQR) body mass index (kg/m^2^)	21.7 (18.6–24.5)
Insurance status (%)	
Uninsured	38 (22.9)
Commercial	67 (40.4)
Medicaid	59 (35.5)
Other	1 (0.6)

Race and insurance status do not add up to 100% due to missing data.

*IQR* interquartile range.

**Table 2:** Presenting signs and symptoms of patients.

n = 166			
Symptoms	Number of patients (%)	Signs	Number of patients (%)
Chest pain	119 (71.7)	None	42 (25.3)
Dyspnea	88 (53.0)	Wheezing	31 (18.7)
Cough	64 (38.6)	Crepitus	30 (18.1)
Neck pain	48 (28.9)	Chest tenderness	27 (16.3)
Throat pain	40 (24.1)	Abdominal tenderness	23 (13.9)
Abdominal pain	32 (19.3)	Hamman’s sign	12 (7.2)
Chest tightness	27 (16.3)	Subcutaneous emphysema	10 (6.0)
Fever	19 (11.4)	Fever	2 (1.2)
Vomiting	64 (38.6)	Neck tenderness	5 (3.0)
Dysphagia	15 (9.0)	Chills	1 (0.6)

### Diagnostic evaluations

Every patient underwent a minimum of one imaging study: 93.4% of patients had a chest radiograph, 58.4% had a CT scan, 40.4% had a CT esophagram, and 18.1% had a fluoroscopic examination. The radiologic results from all imaging studies showed 100% of patients had mediastinal air present, 68.7% had subcutaneous emphysema, 21.1% had a pneumothorax, 6.6% had a pneumopericardium, and 1 patient (0.6%) had a pneumorrhachis. Additionally, one patient with SPM secondary to emesis was found to have imaging concerning for esophageal wall defects but no extraluminal contrast was identified. A second patient with SPM secondary to emesis had a non-full thickness esophageal tear and remained hemodynamically stable with no interventions. Patients underwent the following laboratory work: 74.1% of patients had a Complete Blood Count (CBC), 74.7% had a Comprehensive Metabolic Panel/Basic Metabolic Panel (CMP/BMP), 18.1% had a CRP, 7.2% had an Erythrocyte Sedimentation Rate (ESR), and 23.5% had no laboratory work. In those who had a CBC and CRP level measured, the median (interquartile range) WBC count and CRP level were 11,900 (9100–16,400)/μL and 7.5 (1–51) mg/L, respectively.

### Procedures and Interventions

Three patients were intubated secondary to their uncontrolled, underlying conditions: pneumonia with history of a BiPAP dependence, respiratory distress secondary to pneumonia-induced Acute Respiratory Distress Syndrome (ARDS), and status asthmaticus. Two patients had endoscopies without repair. The endoscopies found normal esophageal anatomy in both cases; one of the cases additionally diagnosed gastroparesis with the endoscopic results. Six patients underwent a bronchoscopy, and of those, one patient additionally had an esophagoscopy. Of those diagnostic procedures, the only abnormal findings were as follows: mild inflammation in the right lobe (one patient), and tracheal injury without subsequent intervention in an immunocompromised patient who was intubated secondary to viral pneumonia. The patient who underwent both a bronchoscopy and esophagoscopy had a history of marfanoid habitus and had no abnormal results through either procedure. Additionally, one patient had a bedside laryngeal scope exam to evaluate for laryngeal injury as the cause, but no abnormalities were found. Esophageal perforation was not identified in any patients.

### Hospital course

**Figure 1:**
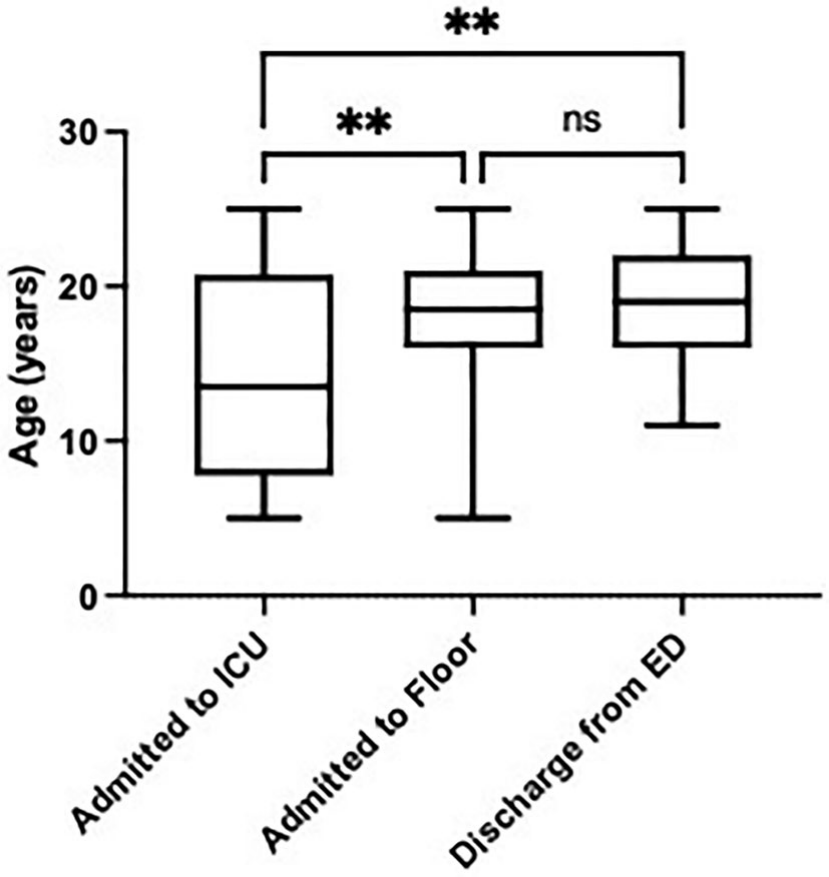
Admission status by age. Box and whiskers plot showing distribution of patient age by admission status. Whiskers show minimum to maximum values. One-way ANOVA was used for comparison. **p < 0.01, *NS* not significant, *ICU* intensive care unit, *ED* emergency department.

84% of patients were admitted to the hospital, indicating only 16% were discharged from the ED. Of those admitted, 15.7% were admitted to Intensive Care Unit (ICU) and 84.3% were admitted to floor. In determining correlation with co-morbidities, patients with asthma made up 68% of the ICU admissions. Additionally, there was found to be a statistically significant difference in patients experiencing an asthma exacerbation being admitted to the ICU versus the floor (*p* = 0.004). Figure 1 illustrates the age correlation with levels of admission. The ICU had the youngest patients of all admissions and discharges from the ED (*p* = 0.003).

Of those discharged, 2 patients returned to the ED within 30 days for unresolving symptoms. One patient returned 3 days later with shortness of breath, however, repeat chest radiographs showed near-complete resolution of pneumomediastinum. They were subsequently discharged from the ED with low-dose Non-steroidal Anti-inflammatory Drugs (NSAIDs). The other patient was initially discharged with spontaneous pneumomediastinum secondary to an asthma exacerbation. They returned 2 days later to the ED with worsening shortness of breath secondary to their asthma exacerbation, admitted to the floor, and then left against medical advice. No patients became septic during the hospital course. No mortalities or morbidities were seen in any of the cases.

For those that were admitted to the hospital, the average length of hospital stay was 2.45 days. 4 out of 163 patients experienced a recurrence of spontaneous pneumomediastinum. The range of days from hospital discharge to the subsequent visit for recurrence was 179–1784 days (5.9 months–4.9 years) with a mean of 875.5 days (2.4 years). All patients who had a recurrence were admitted in their initial presentation. One patient experienced a recurrence years later when they were 28 years-old, which was outside the age range of our inclusion criteria, so their recurrence episode was not included in our study.

Medical treatment, both for discharge and admission, primarily consisted of antibiotics for prophylaxis or to treat an underlying infection and analgesics for pain control. In our study, 44% of patients were prescribed antimicrobials (includes antibiotics, antivirals, and antifungals), 35.5% were prescribed opioids, 33.7% were prescribed NSAIDs, 27.1% were prescribed steroids, 31.3% were prescribed inhalers, and 10.2% were given no medication.

### Age-based cohorts

In comparing demographic and presenting information of those 5–14 years-old to those 15 years-old and older, younger patients were found to have a more even sex distribution (56% male vs. 77% male in ≥ 15 years old patients), higher proportion of asthma, Medicaid insurance, gradual onset, and dyspnea as the presenting feature. Additionally, younger patients were less likely to receive a CT scan (see Table 3).

**Table 3:** Comparison of young and older patients in presentation and imaging.

	< 15 years-old n = 32	≥ 15 years-old n = 134	*P* value
Male	18 (56)	103 (77)	0.026
Medicaid	12 (66)	38 (28)	0.0002
Gradual onset	19 (59)	41 (31)	0.004
Dyspnea	24 (75)	64 (48)	0.006
CT scan	8 (25)	89 (66)	< 0.0001
Asthma exacerbation at onset	7 (22)	9 (7)	0.017
History of vomiting	7 (22)	57 (43)	0.042

Number of patients (%) is shown. *P* value was calculated using chi square test.

### Admission versus discharge

Our analysis included comparing the demographic and presenting features of patients admitted to the hospital (floor or ICU) with those who were discharged from the ED. We found no differences in demographic or presenting features between these cohorts. Additionally, those who were discharged from the ED and those who were admitted were equally likely have a CT scan performed (Table 4).

**Table 4:** Demographics and presenting features of patients who were admitted and discharged from the emergency department.

	Admitted n = 140	Discharged n = 26	*P* value
Male sex, n (%)	104 (74)	17 (65)	0.346
Age in years, median (IQR)	18 (15–21)	19 (16–22)	0.094
Race, n (%)			
Black	23 (17)	2 (8)	0.374
Hispanic	14 (10)	1 (4)	0.470
White	93 (66)	19 (73)	0.650
Insurance, n (%)			
Commercial	53 (38)	14 (54)	0.135
Medicaid	51 (37)	8 (31)	0.660
Uninsured	34 (24)	4 (15)	0.447
Acute onset, n (%)	86 (61)	20 (77)	0.182
Asthma, n (%)	55 (39)	9 (35)	0.827
Symptoms, n (%)			
Chest pain	102 (73)	17 (65)	0.480
Wheezing	26 (19)	5 (19)	> 0.999
Subcutaneous emphysema	10 (7)	0 (0)	0.365
Crepitus	29 (21)	1 (4)	0.050
Hamman’s crunch	11 (8)	1 (4)	0.694
No significant findings on physical exam	27 (19)	15 (58)	0.0001
Imaging, n (%)			
Underwent CT scan	85 (61)	12 (46)	0.196
Pneumothorax	34 (24)	1 (43)	0.018
Pneumopericardium	11 (8)	0 (0)	0.216
Pneumoperitoneum	4 (3)	1 (4)	0.578
Vital signs, median (IQR)			
Heart rate (bpm)	92 (74–115)	85 (69–103.3)	0.022
Respiratory rate (bpm)	20 (16–22)	18 (16–20)	0.001
Temperature (°C)	36.8 (36.7–37.1)	36.7 (36.5–36.9)	0.244
O_2_ saturation (%)	98 (96–99)	99 (98–100)	0.010

*IQR* interquartile range, *CT* computed tomography, *bpm* beats per minute.

As displayed in Table 4, in comparing presenting vital signs of overall admissions and discharges from the ED, those admitted had significantly higher heart rate, respiratory rate, and a lower O_2_ saturation. Next, we sub-analyzed presenting vital signs of patients admitted to the floor with patients discharged from the ED. The heart rate and respiratory rate difference between the two groups were no longer of statistical significance. The O_2_ saturation was lower in patients who were admitted to the floor then those who were discharged from the ED (*p* = 0.026), however, the difference between 97.6% and 98.6% O2 saturation, respectively, is not of clinical significance (Table 5).

**Table 5:** Comparison of presenting vital signs in floor admissions and discharges from ED.

	Floor admissions n = 118	Discharge n = 26	*P* value
Mean (SD) age (years)	18.0 (4.6)	19.0 (3.6)	0.311
Mean (SD) heart rate (bpm)	91.4 (24.1)	86.0 (19.3)	0.222
Mean (SD) respiratory rate (bpm)	19.0 (4.2)	18.1 (2.6)	0.160
Mean (SD) O_2_ saturation (%)	36.9 (0.4)	36.7 (0.4)	0.041
Mean (SD) O_2_ saturation (%)	97.6 (2.5)	98.6 (1.9)	0.026

*P* value was determined using Welch’s t test.

*SD* standard deviation, *bpm* beats per minute.

## Discussion

The results of our study suggest that although the majority of patients with spontaneous pneumomediastinum undergo an unremarkable clinical course, a large number of patients are still admitted and subjected to an extensive workup, creating unnecessary cost and burden to the patients and healthcare system. Their workup included chest radiographs followed by CT imaging, however, the additional imaging did not impact clinical decision making. This applies to those without traumatic or iatrogenic etiologies, as excluded based on our study criteria. Additionally, this study found that patients without pre-existing pulmonary co-morbidities, co-existing infections, or high suspicion of Boerhaave Syndrome did not have any outcomes that required intervention; their admissions were primarily for observation. The patients who underwent admission and were placed in the ICU necessitated escalation in care secondary to their known pulmonary pathologies, not because of spontaneous pneumomediastinum. Spontaneous pneumomediastinum in these cases were incidental complications of their underlying pathology rather than the primary concern for their admission. Our study highlights the over-utilization of imaging in patients with suspected spontaneous pneumomediastinum and escalation in care despite no clinical evidence of its necessity.

Overall, the results of our study show no difference in demographic features, presenting features, complications, or outcomes between those admitted to the floor and those that were discharged from ED. This indicates that there was no clear clinical algorithm used to determine who required admission and who could be discharged from the ED. Rather, it was determined by the physicians on shift at the time, creating inconsistency and variability in care and therefore associated costs. We argue that spontaneous pneumomediastinum is a benign condition that requires treatment of the underlying pathology, rather than the spontaneous pneumomediastinum itself. In all the cases, the only significant complications identified were secondary to their underlying conditions. These patients were gravely ill independent of their SPM diagnosis. Scope of treatment in SPM cases should not consider risk of recurrence as they are incredibly rare and temporally distant from the initial SPM episode. Similarly, to our study, Fitzwater et al. retrospective review of pediatric SPM noted only 3 recurrences that all occurred over a year later. The recurrences were all in asthmatics and had no complications^7^. Additionally, our two patients that returned to the ED after initial ED discharge further contribute to our conclusions: one patient was experiencing a continued asthma exacerbation and the second patient was found to have near-complete resolution of SPM on chest radiograph and subsequently discharged from the ED with NSAIDs. Our study has shown that patients with uncontrolled asthma necessitate escalation in care in an inpatient setting, regardless of their SPM. The second patient was found to have radiograph evidence of self-resolution of SPM within 3 days of initial ED visit. This patient exemplifies the benign, short course of SPM that warrants conservative treatment, which in this case was solely over-the-counter NSAIDs. Takada et al. conducted a literature review on 17 spontaneous pneumomediastinum studies (336 cases) and concluded that 2-day inpatient observation without antibiotics, limitation of oral intake, or invasive examination should be conducted in spontaneous pneumomediastinum. However, in all 17 studies examined, the only serious complication found was a tension pneumomediastinum secondary to mechanical ventilation, which is considered a secondary pneumomediastinum^8^. This complication is not considered spontaneous pneumomediastinum as it is secondary to iatrogenic causes. Therefore, they have no significant data to support their clinical conclusion that inpatient monitoring is necessary. Additionally, recent retrospective review reports the utility of outpatient management for SPM with most of their patients were followed in an outpatient setting with no complications seen^9^. To that end, Macia et. al deemed significant complications secondary to SPM as “virtually nonexistent”^10^. Like Takada et al., we found no evidence of adverse outcomes or complications that necessitate inpatient observation for low-risk patients.

Because of the delicacy of cardio-pulmonary anatomy, clinicians gravitate toward cautious over-imaging of spontaneous pneumomediastinum in concern of missing a tracheal or esophageal perforation. Almost all patients, 93%, had a chest radiograph and 70% had a CT (CT esophagram or CT scan). Additionally, we found that admitted patients and discharged patients from the ED had an equal likelihood of having a CT scan performed with the vast majority of imaging results indicating an isolated SPM with subcutaneous emphysema. Yet over 80% of patients were admitted to the hospital. This data illustrates two points: (1) there was no clear criteria for patient qualifiers for advanced imaging and (2) the CT imaging did not make a difference in clinical decisions as most patients were admitted regardless of the radiologic results. Noorbakhsh et al. found that 75% of their pediatric patients underwent additional imaging after the diagnosis of SPM was made with initial imaging study. Similarly, the additional imaging did not provide further diagnostic information to guide treatment^11^. To further emphasize the over-utilization of imaging studies, a recent retrospective study found that 31% of their patients underwent esophagrams in concern for esophageal perforations, however, they all yielded negative findings^12^. Similarly, our study found no evidence of esophageal perforation in any of other patients, but had 2 patients with incomplete esophageal tears. Our 2 patients remained hemodynamically stable without any complication or intervention until discharge or leaving against medical advice. Bakhos et al. argues that clinicians over-emphasize the overlap in clinical presentation of SPM and pneumomediastinum secondary to esophageal perforation. Patients with pneumomediastinum secondary to esophageal perforations were significantly older (mean age of 58 years old), higher heart rate, higher BMI, greater leukocytosis, and were more likely to have evidence of pleural effusion and atelectasis on imaging^13^. Additionally, Forshaw et al. found that those who had pneumomediastinum secondary to Boerhaave syndrome presented with signs of septic shock and necessitated early resuscitation^6^. This presentation is vastly different from SPM without Boerhaave syndrome, as further emphasized by our study, that those with SPM without Boerhaave syndrome presented hemodynamically stable at the ED. This does not include those in diabetic ketoacidosis secondary to emesis. In our study, 64 patients presented with emesis and underwent multiple imaging studies with no significant findings that warranted intervention. These findings provide evidence to support that SPM secondary to emesis does not require additional imaging beyond a chest radiograph if there is no evidence of systemic toxicity or chest radiograph findings with suspicion for esophageal perforation.

In our study, we found that there was a significant correlation between ICU admission, age, and asthma. Those with admitted to the ICU were the youngest of our cohort and 68% had asthma. Additionally, we found that younger patients (5- to 14-years-old) were significantly more likely to have Medicaid insurance than older patients. In using public insurance as a proxy for low socioeconomic status, this illustrates a potential discrepancy in access to primary care and therefore appropriate asthma management and exacerbation prevention. This disparity is well-documented in literature stating that children with asthma and Medicaid insurance have a substantially higher risk of repeat asthma exacerbations requiring hospitalization^14^. Of our cases of SPM in children with Medicaid insurance and ICU admission, SPM was an incidental finding secondary to the severe asthma exacerbations that warranted their escalation in care. Our findings are supportive of the argument that those of lower socioeconomic households, despite having Medicaid insurance, are not provided with access to quality care and therefore require continual hospitalizations to manage their asthma.

We understand that there are inherent limitations to retrospective studies given they are reliant on electronic medical records for data and cannot control their accuracy. In addition, we cannot account for patients who went to a different healthcare system for their SPM. However, most of our patients had healthcare appointments after the SPM episode in our health system with no mention of SPM, thereby giving us reason to believe they experienced no adverse sequelae. Additionally, we acknowledge that because our data is from a single center with a small number of cases, it may not be representative of the field. However, our study is the largest study on spontaneous pneumomediastinum in pediatrics and young adults to date and provides valuable information to better patient care and decrease associated costs with evidence-based medicine.

In conclusion, we have found that spontaneous pneumomediastinum is a benign, self-resolving entity whose management should focus on addressing the underlying cause (if present), rather than the spontaneous pneumomediastinum itself. Based on our study, in those without an underlying condition nor displaying signs of pending or present hemodynamic instability, ED discharge with outpatient follow-up after a brief observational period and patient education of concerning symptoms requiring immediate care (worsening shortness of breath, purple/blue skin discoloration, etc.) is the appropriate management. Providers are often in fear of serious sequelae of unmonitored SPM, however, none of our cases required intervention nor experienced morbidities secondary to SPM. Imaging beyond an initial chest radiograph is unnecessary in patients who display no signs of toxicity or have no underlying clinical condition that requires more aggressive attention.

## Data Availability

The deidentified dataset analyzed in this study is available from the corresponding author on reasonable request.

## References

[1] Langwieler TE, Steffani KD, Bogoevski DP, Mann O, Izbicki JR (2004). Spontaneous pneumomediastinum. Ann. Thorac. Surg..

[2] Kouritas VK (2015). Pneumomediastinum. J. Thorac. Dis..

[3] Talwar A, Esquire A, Sahni S, Verma S, Grullon J, Patel P (2013). Spontaneous pneumomediastinum: Time for consensus. N. Am. J. Med. Sci..

[4] Bullaro FM, Bartoletti SC (2007). Spontaneous pneumomediastinum in children. Pediatr. Emerg. Care.

[5] Damore DTM, Dayan PSM (2001). Medical causes of pneumomediastinum in children. Clin. Pediatr. (Phila.).

[6] Forshaw MJ, Khan AZ, Strauss DC, Botha AJ, Mason RC (2007). Vomiting-induced pneumomediastinum and subcutaneous emphysema does not always indicate Boerhaave’s syndrome: Report of six cases. Surg. Today.

[7] Fitzwater JW, Silva NN, Knight CG, Malvezzi L, Ramos-Irizarry C, Burnweit CA (2015). Management of spontaneous pneumomediastinum in children. J. Pediatr. Surg..

[8] Takada K (2009). Spontaneous pneumomediastinum: An algorithm for diagnosis and management. Ther. Adv. Respir. Dis..

[9] Ebina M, Inoue A, Takaba A, Ariyoshi K (2017). Management of spontaneous pneumomediastinum: Are hospitalization and prophylactic antibiotics needed?. Am. J. Emerg. Med..

[10] Macia I (2007). Spontaneous pneumomediastinum: 41 cases. Eur. J. Cardio-Thorac. Surg..

[11] Noorbakhsh KA (2021). Management and outcomes of spontaneous pneumomediastinum in children. Pediatr. Emerg. Care.

[12] Dougherty D, Meyer KM, Thompson AR, Speck KE (2022). Pediatric pneumomediastinum: Symptom-based management. J. Pediatr. Surg..

[13] Bakhos CT, Pupovac SS, Ata A, Fantauzzi JP, Fabian T (2014). Spontaneous pneumomediastinum: An extensive workup is not required. J. Am. Coll. Surg..

[14] Camargo CA, Ramachandran S, Ryskina KL, Lewis BE, Legorreta AP (2007). Association between common asthma therapies and recurrent asthma exacerbations in children enrolled in a state Medicaid plan. Am. J. Health-Syst. Pharm..

